# Identification of sulfakinin receptor regulating feeding behavior and hemolymph trehalose homeostasis in the silkworm, *Bombyx mori*

**DOI:** 10.1038/s41598-024-65177-z

**Published:** 2024-06-20

**Authors:** Jiajing Lan, Qi Wu, Nan Huang, Hong Zhang, Yuanfei Yang, Linjie Chen, Naiming Zhou, Xiaobai He

**Affiliations:** 1 School of Laboratory Medicine and Bioengineering, Hangzhou Medical College, Hangzhou, 311399 China; 2 College of Biotechnology, Jiangsu University of Science and Technology, Zhenjiang, 212018 Jiangsu China; 3 Department of Clinical Laboratory, The First People’s Hospital of Lin’an District, Hangzhou, 311399 Zhejiang China; 4 Institute of Biochemistry, College of Life Sciences, Zijingang Campus, Zhejiang University, Hangzhou, 310058 Zhejiang China

**Keywords:** *Bombyx mori*, Sulfakinin, G protein-coupled receptor, Satiety factor, Trehalose, Cell biology, Zoology

## Abstract

Feeding behavior, the most fundamental physiological activity, is controlled by two opposing groups of factors, orexigenic and anorexigenic factors. The sulfakinin family, an insect analogue of the mammalian satiety factor cholecystokinin (CCK), has been shown to suppress food intake in various insects. Nevertheless, the mechanisms through which sulfakinin regulates feeding behavior remain a biological question. This study aimed to elucidate the signaling pathway mediated by the anorexigenic peptide sulfakinin in *Bombyx mori*. We identified the *Bombyx mori* neuropeptide G protein-coupled receptor A9 (BNGR-A9) as the receptor for sulfakinin through functional assays. Stimulation with sulfakinin triggered a swift increase in intracellular IP3, Ca^2+^, and a notable enhancement of ERK1/2 phosphorylation, in a manner sensitive to a Gα_q_-specific inhibitor. Treatment with synthetic sulfakinin resulted in decreased food consumption and average body weight. Additionally, administering synthetic sulfakinin to silkworms significantly elevated hemolymph trehalose levels, an effect markedly reduced by pre-treatment with BNGR-A9 dsRNA. Consequently, our findings establish the sulfakinin/BNGR-A9 signaling pathway as a critical regulator of feeding behavior and hemolymph trehalose homeostasis in *Bombyx mori*, highlighting its roles in the negative control of food intake and the positive regulation of energy balance.

## Introduction

Regulating feeding in *Bombyx mori* is crucial for optimizing silk production, as well as maintaining population density and health. However, we still do not fully understand the molecular mechanisms behind feeding regulation in this species. Sulfakinins (SKs), insect neuropeptides akin to the vertebrate gastrin/cholecystokinins involved in feeding regulation, have been shown to play a regulatory role in various insect species, including the desert locust *Schistocerca gregaria*, the beetle *Tribolium castaneum* and *Dendroctonus armandi*^1–3^. The role of *Bombyx mori* SKs and their receptors in feeding regulation, however, remains to be elucidated.

SKs were first isolated from the head extract of the cockroach *Leucopaea maderae*^4,5^ and subsequently characterized in various species, including the cockroach *Periplaneta americana*^6^, the locust *Locusta migratoria*^7^, the flesh fly *Neobellieria bullata*^8^, and the fruit fly *Drosophila melanogaster*^9^. SKs in insects exhibit varied physiological roles across different species. In *Locusta migratoria,* SKs have been reported to enhance the release of primary digestive enzymes within the insect gut^10,11^. Additionally, SKs also elevate heart contraction frequencies in *Drosophila melanogaster*^12,13^, and act as modulators of odor preference, locomotion^14,15^, and metabolism^16^. Nevertheless, SKs are most renowned for their role as a satiety factor in regulating feeding behavior. SKs have been shown to reduce meal sizes in various insects upon injection, such as the blowfly *Phormia regina*, and beetles *Zophobas atratus* and *Tribolium castaneum*^1,2,17–20^.

Sharing structural similarities with vertebrate CCKs, these identified SKs possess a conserved C-terminal hexapeptide (Y(SO_3_H)GHM/L)RFamide) and a sulfated tyrosine residue critical for their receptor binding and biological function across several insect species^5,17–20^. Experiments with synthetic non-sulfated Leucosulfakinins (LSKs) revealed no myotropic activity on the cockroach hindgut, in contrast to sulfated LSKs, which significantly increased spontaneous hindgut contractions^5^. Similarly, in the desert locust *Schistocerca gregaria* and brown planthopper *Nilaparvata lugens*, the full efficacy and potency of SKs in reducing feeding also requires a Tyr(SO_3_H) moiety^1,20^.

Insect SKs mediate their effects via G protein-coupled receptors (GPCRs) located on the cell surface membrane. The *Drosophila* SK receptor (DSK-R1, also known as CCKLR-17D3) emerged as the first cloned and characterized receptor for insect SKs, displaying high affinity for tyrosine-sulfated SK (sSK) and low affinity for nonsulfated SK (nsSK)^21^. Another receptor, CCKLR-17D1, identified in *Drosophila*, exclusively responds to sSKs, underscoring the critical role of the sulfated tyrosine residue in SKs for receptor interaction and activity^14^. This specificity for sSKs was also observed in the beetle *Tribolium castaneum*. Cell-based receptor assays demonstrated that sulfated SKs more effectively activated the SK receptors in *Tribolium castaneum* compared to their nonsulfated counterparts, reinforcing the significance of sulfation for receptor activation. Furthermore, knockdown experiments targeting the sk, skr1, or skr2 genes in *Tribolium castaneum* led to increased food consumption, highlighting the regulatory role of these genes in feeding behavior^2,19^.

In this study, we describe the cloning of cDNA for the *Bombyx mori* SK receptor, BNGR-A9. Through a series of assays, including a CRE-driven luciferase reporter system for cAMP measurement, a Fura-2 AM-based calcium mobilization assay, and a receptor internalization assay, we determined that BNGR-A9 serves as a specific receptor for *Bombyx* SKs. Further support from expression profiling and in vivo experiments demonstrates that the SK/BNGR-A9 signaling pathway plays a crucial role in regulating food intake and hemolymph trehalose levels in *Bombyx mori*. Our results elucidate the signaling mechanisms underlying silkworm feeding behavior and highlight a potential molecular target for controlling Lepidoptera populations.

## Results

### Cloning and comparative analysis of BNGR-A9 and other SK receptors sequences

**Figure 1:**
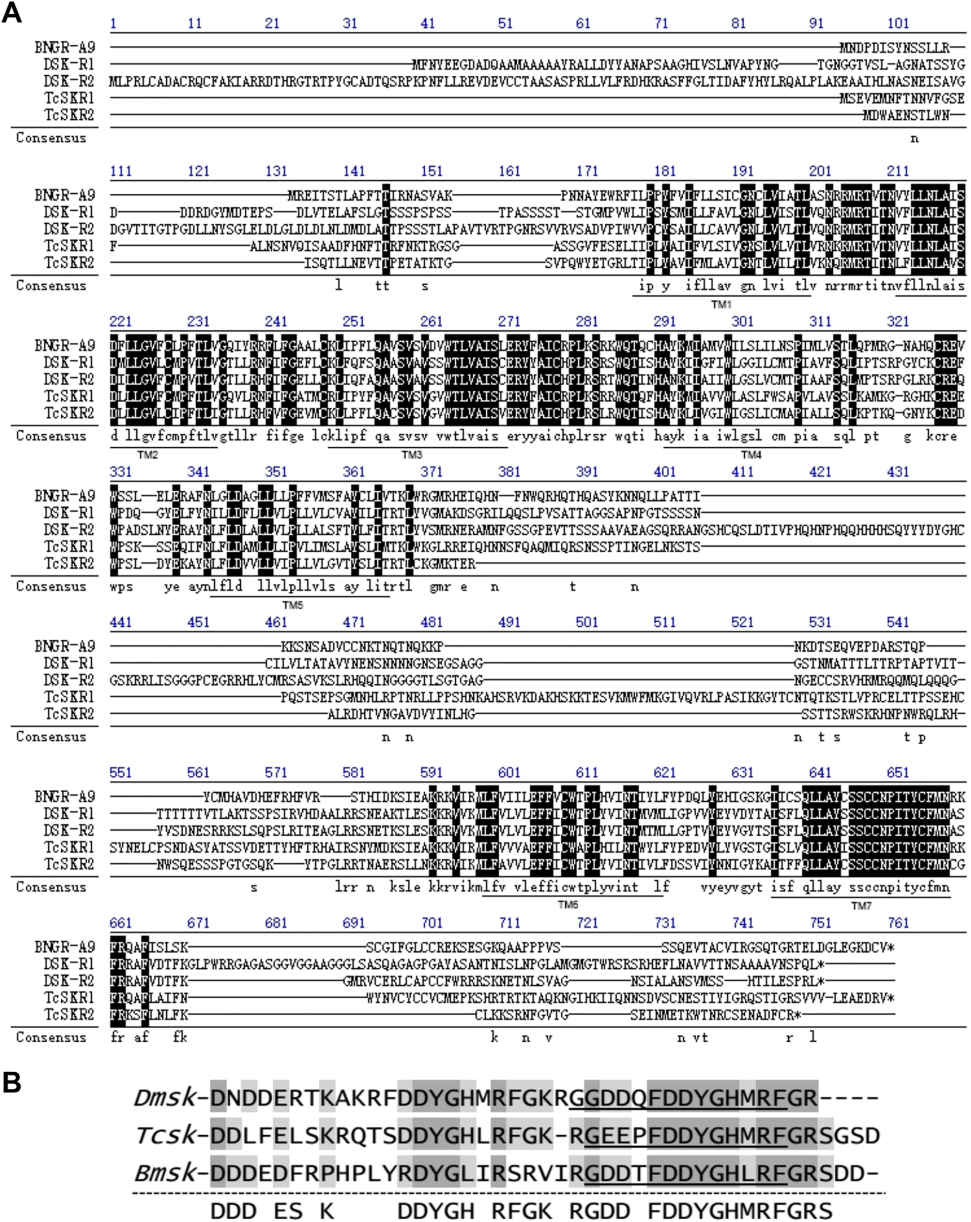
Sequences of BNGR-A9 and the *Bombyx mori* SK. (**A**) Comparison of amino acid sequences of BNGR-A9 from four characterized insect SK receptors. The amino acid sequence of BNGR-A9 are aligned with *Drosophila melanogaster* SK receptors DSK-R1 and DSK-R2, and *Tribolium castaneum* SK receptors TcSKR1 and TcSKR2. Sequence identities are indicated below the sequence. Seven putative transmembrane regions (TM1-TM7) are displayed as indicated. (**B**) The sequence alignment of the predicted *Bombyx mori* SK prepropeptide with *Drosophila melanogaster* and *Tribolium castaneum* SK. The predicted mature peptides are indicated by underline.

BNGR-A9 was identified as a potential SK receptor in *Bombyx mori* through genomic and phylogenetic analyses^22,23^. In this research, we successfully cloned the full-length cDNA of BNGR-A9 (GenBank: NM_001134272.1) from *Bombyx mori* larval brain tissue via RT-PCR. The BNGR-A9 protein comprises 464 amino acids, featuring the characteristic seven hydrophobic domains typical of GPCRs. Sequence comparison showed BNGR-A9 shares sequence identities of 31.6%, 29%, 42.8%, and 40.8% with DSK-R1, DSK-R2, TcSKR-1, and TcSKR-2 insect SK receptors, respectively (Fig. 1A).

SK family peptides typically end with the motif -Y(SO3H)GHM/LRF-NH2. Within the *Bombyx mori* genome, we identified a transcript (GenBank: NM_001130882) coding for a SK precursor. Analysis of conserved cleavage sites revealed a single mature SK peptide featuring -Lys-Arg- or -Arg- and an amidation site (GDDTFDD^8^YGHLRF-NH2). This mature peptide shares nine residues with SKs from other insects (Fig. 1B). Previous studies have shown SK tyrosine (-^8^Tyr-) can be sulfated or nonsulfated ^6,24^. Consequently, we synthesized both sulfated (BmsSK) and nonsulfated (BmnsSK) versions of *Bombyx mori* SK for further experiments.

### Expression of *Bombyx* SK and BNGR-A9 mRNA across tissues in *Bombyxmori*

**Figure 2:**
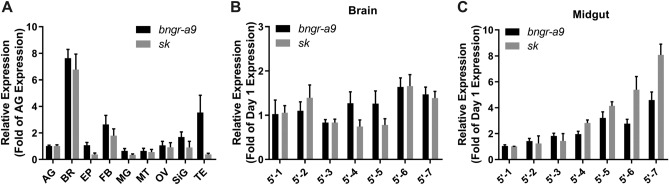
Expression profile of *sk* and *bngr-a9* in *Bombyx mori*. (**A**) Transcript profiles of *sk* and *bngr-a9* in tissues of 2nd-day larvae of the fifth instar were analyzed by qRT-PCR and normalized to the geometric mean of reference genes GAPDH and Actin A3. Abbreviations: AG, abdominal ganglion; BR, brain; EP, epidermis; FB, fat body; MG, midgut; MT, Malpighian tube; OV, ovary; SiG, silk gland; TE, testis. The data displayed as the folds of *bngr-a9* or *sk* in AG, respectively. (**B**) Kinetic expression level of *bngr-a9* and *sk* in Brain of fifth instar larvae. (**C**) Kinetic expression level of *bngr-a9* and *sk* in the Midgut of fifth instar larvae.Data were normalized to the geometric mean of reference genes GAPDH and Actin A3 and displayed as the folds of the first-day expression level of *bngr-a9* and *sk*, respectively. All data were taken from at least three independent experiments.

To explore the distribution of SK and BNGR-A9 mRNA, we conducted qRT-PCR on various tissues of fifth instar silkworm larvae (Fig. 2). The findings revealed the highest expression of *sk* in the brain, consistent with the localization of its potential receptor, *bngr-a9*. Notably, lower expression levels of *bngr-a9* were also found in the testis and fat body, whereas in other tissues, both *sk* and *bngr-a9* mRNA were nearly undetectable (Fig. 2A).

Additionally, we examined the expression patterns of these genes in the brain and midgut during the fifth instar stage. Both genes maintained constant expression in the brain throughout this stage (Fig. 2B). However, in the midgut, both genes gradually increased their expression starting from the third day of the fifth instar stage, peaking on the last day when *Bombyx mori* completely stops feeding (Fig. 2C). These findings not only suggest the role of the SK signaling pathway in regulating food intake but also hint at its involvement in various physiological processes across different tissues.

### Identification of BNGR-A9 as the functional *Bombyx* mori sulfakinins receptor

**Figure 3:**
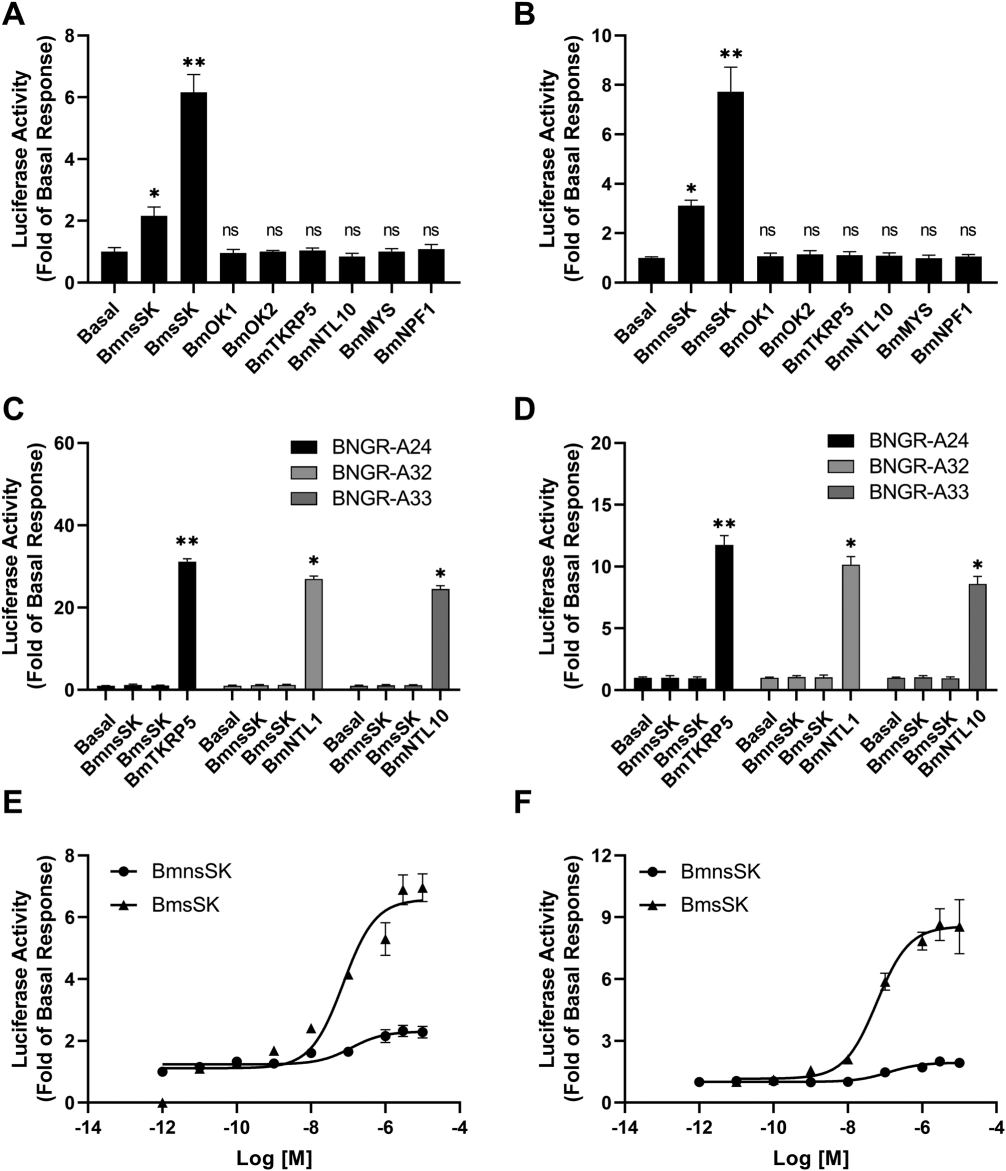
BNGR-A9 is a specific *Bombyx mori* sulfakinin peptides receptor. HEK293 cells (**A**) or BmN cells (**B**) transfected with BNGR-A9 and the corresponding reporter gene pCRE-Luc were treated with 1 μM of different *Bombyx mori* neuropeptides for 4 h, and responses were normalized against the luciferase activity of Basal. Abbreviations: nsSK, nonsulfated-sulfakinin; sSK, sulfated-sulfakinin; TKRP, tachykinin-related peptide; NTL, natalisin; OK, orcokinin; MYS, myosuppressin; NPF1, neuropeptide F1. (**C**) HEK293 cells transiently transfected with BNGR-A24, BNGR-A32 or BNGR-A33 and the reporter gene pCRE-Luc, and then treated with 1 μM of *Bombyx* SKs or their corresponding ligands, followed by detection of luciferase activities. (**D**) BmN cells expressing BNGR-A24, BNGR-A32 or BNGR-A33 and the reporter gene pCRE-Luc, were treated with 1 μM *Bombyx* SKs or their corresponding ligands for 4 h, followed by detection of luciferase activities. Data were analyzed by using two-way ANOVA with multiple comparisons to the control response (basal column) (*p < 0.05; **p < 0.01). (**E** and **F**) Dose–response curves of HEK293 cells (**E**) and BmN cells (**F**) stably expressing BNGR-A9 and the corresponding reporter gene pCRE-Luc treated with different concentration of *Bombyx* SKs. All data were taken from at least three independent experiments.

The qRT-PCR analyses revealed a close correlation between the expression profiles of *sk* and *bngr-a9*, suggesting a strong likelihood that BNGR-A9 serves as a receptor for *Bombyx* SK. Next, we aimed to verify if BNGR-A9 functions as the receptor for *Bombyx* SKs. We used a CRE-driven luciferase reporter assay to indirectly measure intracellular cAMP production. Testing various *Bombyx* neuropeptides in a heterologous expression system, we found that only BmsSK and BmnsSK could activate BNGR-A9 in HEK293 and BmN cells (Fig. 3A and B), regardless of the presence of forskolin (Fig. S1). Other *Bombyx* mori neuropeptides, including neuropeptide F, myosuppresin from the RF-NH2 family, and peptides such as TKRP5 (tachykinin-related peptide 5), OK (orcokinin), or NTL (natalisin), did not significantly activate BNGR-A9 expressing cells.

Additionally, we tested *Bombyx* SKs on evolutionary closely related receptors BNGR-A24, -A32, and -A33, which showed no response, confirming BNGR-A9's specificity for *Bombyx* SKs (Fig. 3C and D). CRE-driven luciferase reporter assay designed to measure cAMP levels, which is a common method to study the activation of Gα_s_-coupled receptors. Interestingly, the activation effect of *Bombyx* SKs on luciferase activity was lower than that previously reported for Gα_s_-coupled receptors such as AKHR or BNGR-A24^25,26^. This suggests that BNGR-A9 may not strongly couple with Gα_s_ and that Gα_q_-coupled signaling might predominantly mediate BNGR-A9 activation.

### Sulfated Sulfakinin is more effective at activating BNGR-A9 than nonsulfated Sulfakinin

To examine the impact of sulfonation (SO_3_H) on the activity of SK peptides towards BNGR-A9, we conducted concentration–response analyses. The results showed that sulfated SK (BmsSK) significantly activated BNGR-A9 at concentrations between 1 and 10 nM, achieving an EC_50_ of 73.3 nM in HEK293 cells and 60.0 nM in BmN cells. In contrast, nonsulfated SK (BmnsSK) required concentrations up to 1μM or even 10μM to activate BNGR-A9, with an EC_50_ of 119.4 nM in HEK293 cells and 125.3 nM in BmN cells (Fig. 3E, F). Additionally, the activation efficacy of BmnsSK was less than 30% of that observed with BmsSK, suggesting BmnsSK functions as a partial agonist for BNGR-A9.

**Figure 4:**
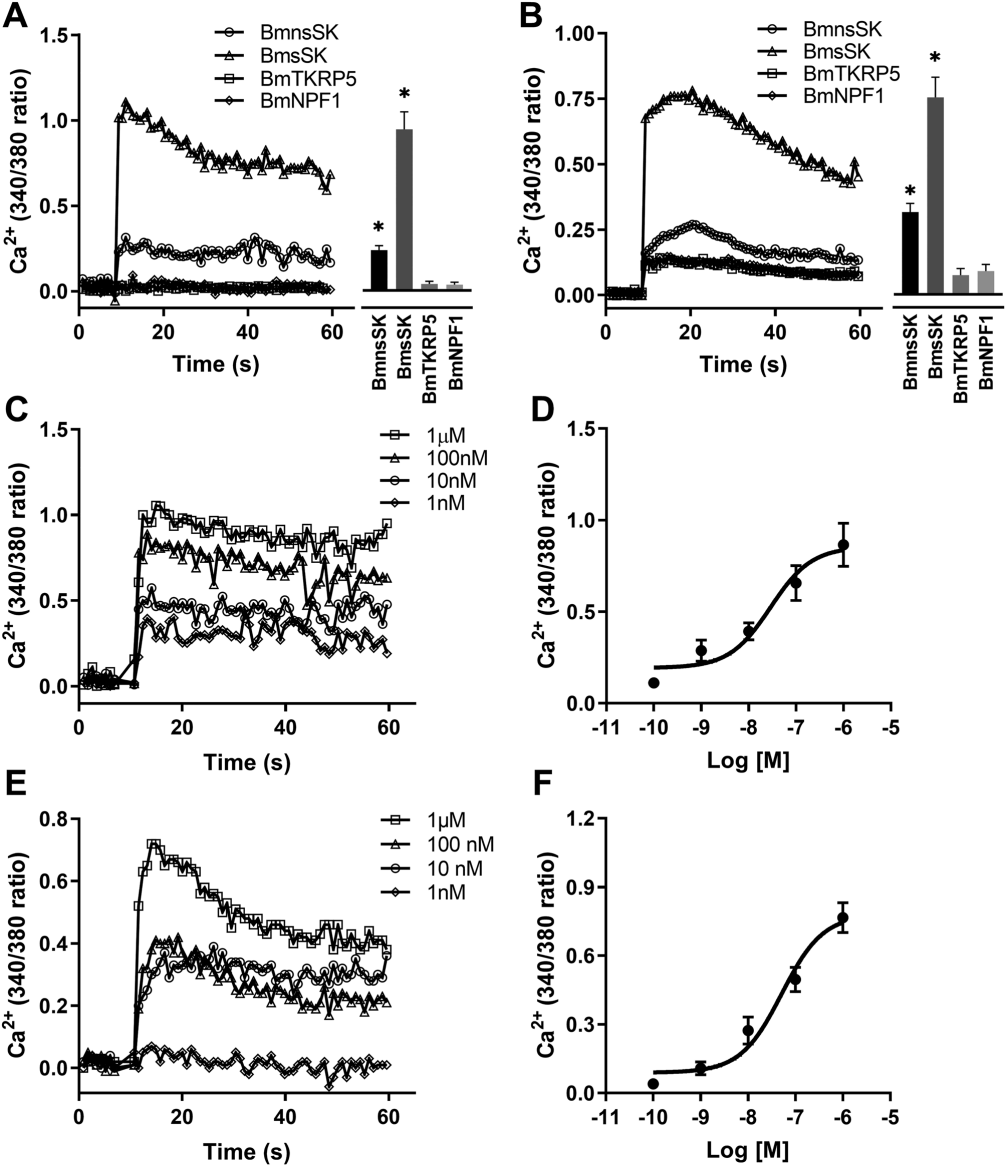
Ca^2+^ mobilization in BNGR-A9 expressing cells in response to *Bombyx* SKs**.** HEK293 (**A**) and BmN (**B**) cells stably expressing BNGR-A9 were exposed to 1 μM different neuropeptides. Cells were preincubated with Fura-2 AM for 1 h, and fluorescence was recorded after stimulation with different *Bombyx* neuropeptides. Curves show the response of neuropeptides subtracted from that of the control. The right panel indicates statistical analysis of three independent experiments. (**C-D**) Calcium mobilization of BNGR-A9-expressing HEK293 cells in response to various concentrations of BmsSK from 1 nM to 1 μM. (**E-F**) Dose–response curve of Ca^2+^ mobilization in response to BmsSK from BNGR-A9 expressing BmN cells. Data indicated statistical analysis of three independent experiments.

**Figure 5:**
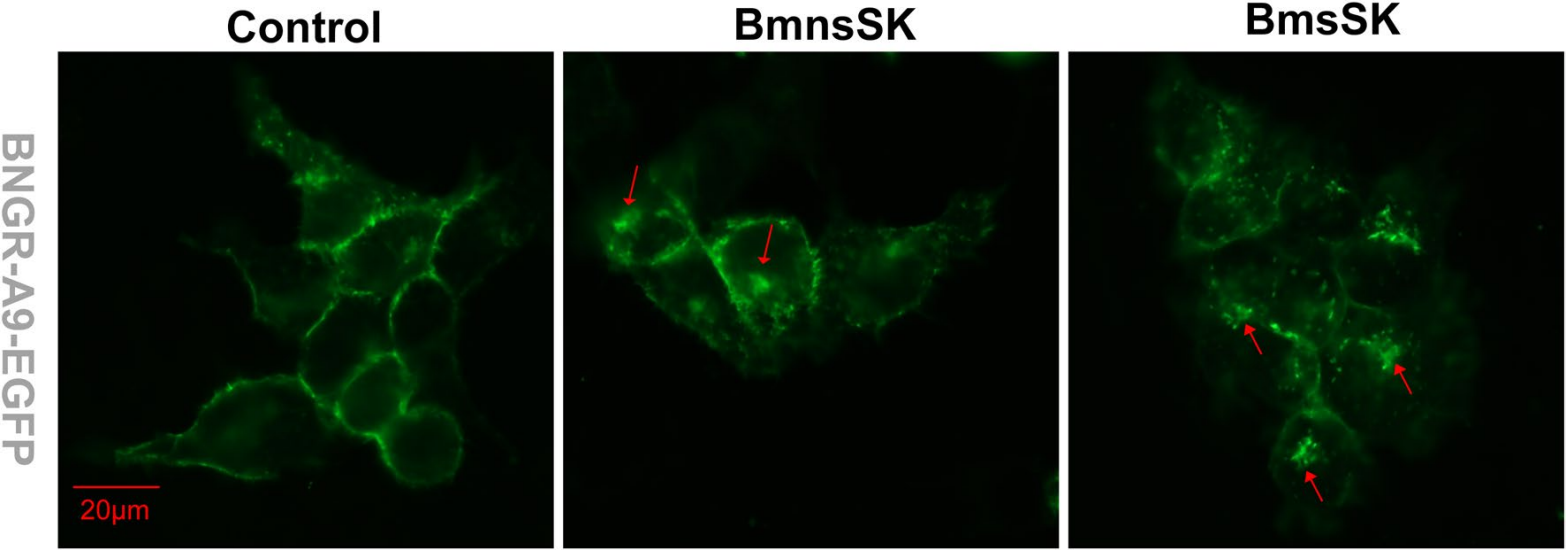
Internalization of BNGR-A9-EGFP induced by *Bombyx* SKs. BNGR-A9-EGFP stably expressing HEK293 cells were incubated with 100 nM BmnsSK or BmsSK for 30min, and examined by Leica fluorescence microscopy. The results are representative of three independent experiments.

Beyond the CRE-driven luciferase assay, the Fura-2 AM-based Ca^2+^ mobilization assay further assessed the signaling activity mediated by SKs through BNGR-A9. As indicated in Fig. 4A and B, both sulfated and nonsulfated SKs at 1 μM concentration triggered responses in BNGR-A9-expressing cells, unlike other tested neuropeptides (tachykinin-related peptide, and neuropeptide F). Dose–response analysis revealed that sulfated SKs stimulated BNGR-A9 at notably lower concentrations (EC50: 29 nM in HEK293 and 51.4 nM in BmN cells) (Fig. 4C–F). Consistent with the luciferase assay findings, BmsSK generated a significantly greater Ca^2+^ response compared to BmnsSK. Furthermore, agonist-induced internalization is a well-characterized phenomenon for most GPCRs. As indicated in Fig. 5, our results showed that BmsSK induced rapid and significant receptor redistribution within the cytoplasm. These findings collectively indicate that BNGR-A9 acts as the specific receptor for *Bombyx* sulfated SK and can be partially activated by nonsulfated SK.

### Activation of BNGR-A9 via Gαq protein-coupled signaling pathway

**Figure 6:**
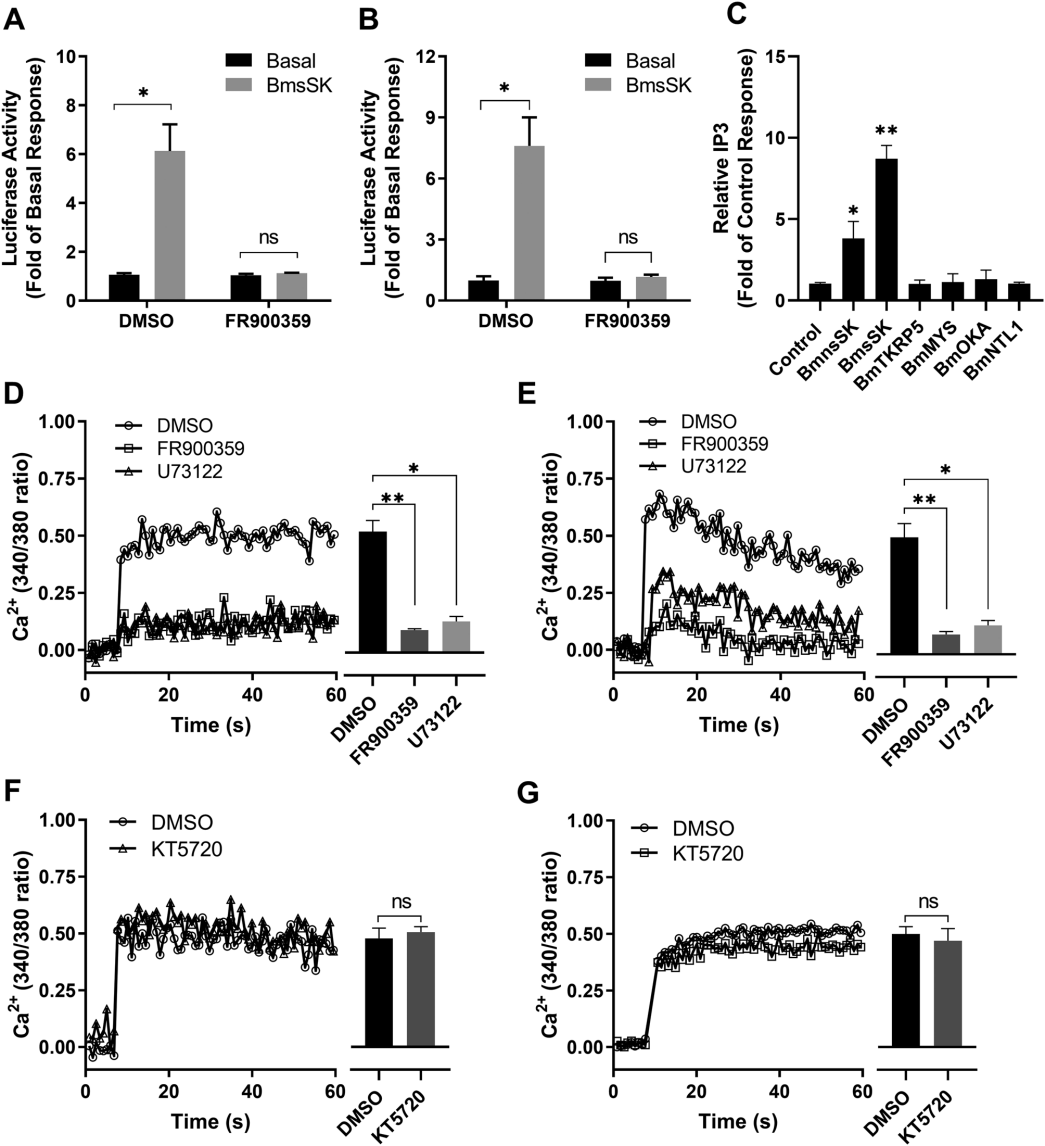
*Bombyx* sulfated-sulfakinin mediated activation of BNGR-A9 via Gα_q_ signaling pathway. (**A** and **B**) Effects of Gα_q_ inhibitor FR900359 (1 μM) on the CRE-driven luciferase activities in BNGR-A9 expressing HEK293 (**A**) and BmN (**B**) cells. BNGR-A9 stable transfected cells were pretreated with FR900359 (1 μM) for 30 min and challenged with 100 nM BmsSK for 4 h. (**C**) Accumulation of IP3 in HEK293 cells expressing BNGR-A9 in response to *Bombyx* neuropeptides was assessed. Cells were pre-treated with serum-free DMEM for 1 h, followed by stimulation with 1 μM of the indicated *Bombyx* neuropeptides for 1 h. The level of IP3 in the supernatant was then determined using the IP3 immunoassay detection kit. (**D**, **E**) Effects of Gα_q_/PLC inhibitors on Flag-BNGR-A9 mediated increase of intracellular Ca^2+^ level. Flag-BNGR-A9 expressing HEK293 (**D**) and BmN (**E**) cells were pretreated with Gα_q_ inhibitorFR900359 (1 μM), U73122 (5 μM) for 30 min, and incubated with Fura-2 AM for 30 min, followed by challenging with 100 nM BmsSK. (**F**, **G**) effects of protein kinase A (PKA) inhibitor KT5720 on BmsSK initiated Ca^2+^ in Flag-BNGR-A9-expressing cells. Flag-BNGR-A9 expressing HEK293 (**F**) and BmN (**G**) cells were pretreated with KT5720 (10 μM) for 30 min prior to the incubation of Fura- 2 AM. After 30 min incubation with Fura-2 AM, the cells were treated with 100 nM BmsSK, and detected with a fluorescence spectrometer. Data indicated statistical analysis of three independent experiments. Data were analyzed by using a Student’s t-test (*p < 0.05; **p < 0.01). All data were taken from at least three independent experiments.

The activation of receptor BNGR-A9 by BmsSK leads to intracellular Ca^2+^ mobilization and CRE-driven luciferase transcription, pointing towards its engagement with a Gα_q_ protein-coupled signaling pathway. To elucidate the specific signaling mechanisms involved, we employed functional assays in conjunction with specific inhibitors. Treatment with FR900359, a Gα_q_-specific inhibitor^27^, significantly reduced the luciferase activity increase induced by BmsSK (Fig. 6A and B). Besides, the intracellular cAMP levels in BNGR-A9-expressing HEK293 cells remained unchanged in response to BmnsSK and BmsSK, as detected using a direct cAMP ELISA kit (Fig. S2). Additionally, intracellular IP3 levels were determined using a direct IP3 ELISA kit, revealing a significant elevation following stimulation with BmnsSK and BmsSK in BNGR-A9-expressing HEK293 cells (Fig. 6C). Similarly, the Ca^2+^ mobilization triggered by BmsSK was completely blocked by FR900359 and the PLC inhibitor U73122, indicating the involvement of these components in BNGR-A9 activation (Fig. 6D and E). However, the PKA inhibitor KT5720 did not affect the BmsSK-induced responses, suggesting a different signaling route (Fig. 6F and G).

**Figure 7:**
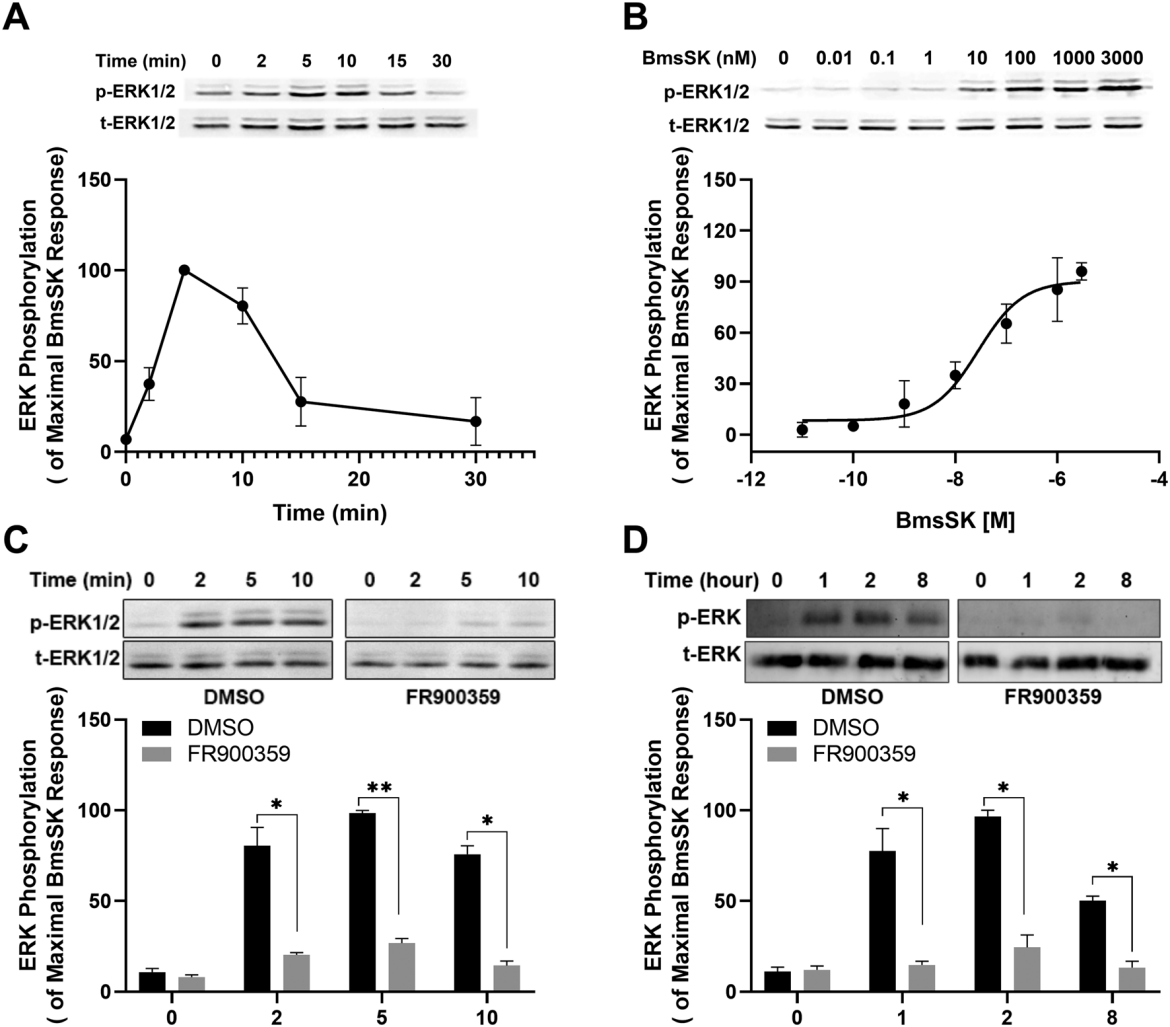
Gα_q_ are involved in BNGR-A9-mediated ERK phosphorylation. (**A** and **B**) BNGR-A9-expressing HEK293 cells were starved using serum-free medium for 1 h, then stimulated with BmsSK under various conditions before harvesting to assess ERK1/2 phosphorylation. (**A**) The cells were treated with 10 nM BmsSK for several time intervals. (**B**) Cells were exposed to varying concentrations of BmsSK for a duration of 5 min. (**C**) Flag-BNGR-A9-expressing HEK293 cells were pretreated with Gα_q_ inhibitor FR900359 (1 μM) or DMSO in serum-free medium for 30 min. The cells were incubated with BmsSK (100 nM) for the indicated time and harvested for immunoblots as described in *Materials and methods*. (**D**) brains dissected from 5th instar silkworm were incubated with Gα_q_ inhibitor FR900359 (1 μM) or DMSO for 30 min, followed by incubation with 100 nM BmsSK for 1, 2, or 8 h. ERK phosphorylation (p-ERK) levels were determined by western blot as described in *Materials and Methods*. Expression of total ERK (t-ERK) was utilized as a loading control to ensure equal protein loading across samples. Data was analyzed using a Student's t-test (*p < 0.05; **p < 0.01). Data represent the mean ± SEM of at least three independent experiments.

Further investigations into ERK phosphorylation in both heterologous and endogenous systems showed that BmsSK activation led to a quick and dose-dependent increase in ERK1/2 phosphorylation, reaching its peak at 5 min in HEK293 cells and 2 h in the silkworm brains (Fig. 7A, B and D). This phosphorylation was notably diminished by pre-incubation with FR900359 (Fig. 7C). Similarly, in silkworm larval brains, BmsSK-induced ERK phosphorylation was inhibited by FR900359, reinforcing the role of Gα_q_ protein-dependent pathways in BNGR-A9 activation (Fig. 7D). These findings indicate the specific activation of BNGR-A9 by *Bombyx* SK through Gα_q_ protein-coupled signaling.

### Sulfakinin-mediated induction of food intake in *Bombyx* mori through BNGR-A9

**Figure 8:**
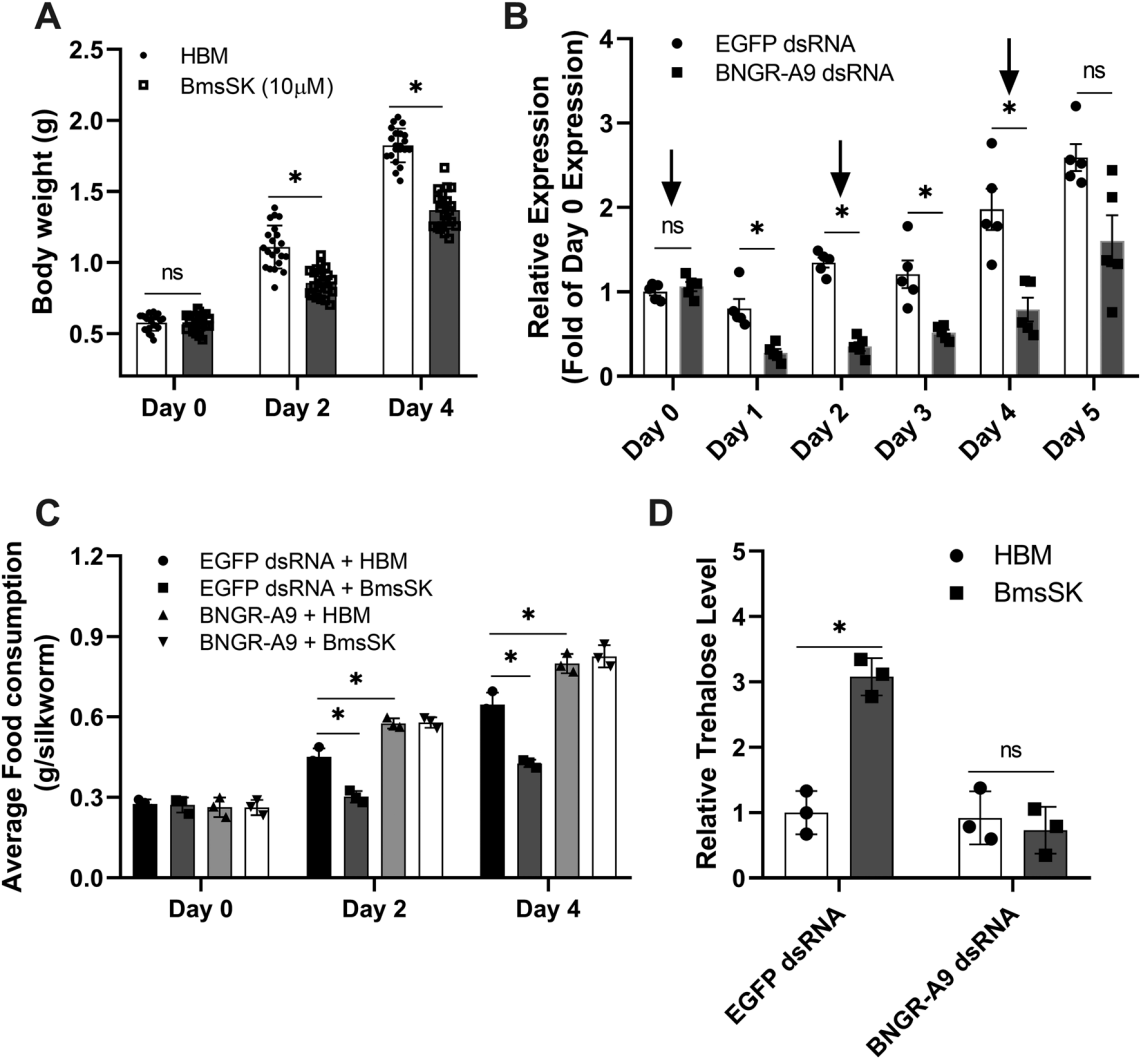
Effects of sulfated- SK and dsRNA-mediated knockdown of BNGR-A9 on feeding behavior of silkworm. (**A**) Effect of *Bombyx* sulfated- SK on silkworm body weight. Larvae were injected with BmsSK (final concentration 10 nM) or an equivalent volume of HBM every day since the second day of 5^th^ instar (Day 0), and body weight was measured every 2 days. (**B**) Effects of dsRNA injection on *bngr-a9* expression in Midgut. BNGR-A9 dsRNA (5 μg) or EGFP dsRNA (5 μg) were injected into larvae of silkworm every other day. *bngr-a9* gene expression level in treated silkworm larvae was determined by qRT-PCR as described in *Materials and Methods*. The data were displayed as fold changes relative to the first-day expression level. (**C**) Effects of BNGR-A9 dsRNA and sulfated-SK on average food intake of silkworm. 24 h after dsRNA injection, synthesized BmsSK (final concentration 10 nM) or HBM were injected into the larvae of silkworm. Food consumption of silkworms was measured every 2 days. (**D**) Effect of dsRNA and sulfated- SK on the trehalose level in the hemolymph. 24 h after dsRNA injection, silkworm larvae were injected with *Bombyx* sulfated- SK (final 10 nM) or the same volume of HBM, then hemolymph was collection 24 h later. All data were taken from at least three independent experiments, and data were analyzed using a Student’s t-test (*p < 0.05).

We then explored the roles of SK and its receptor BNGR-A9 in regulating food intake in *Bombyx mori*. At the beginning of the fifth instar, we injected the silkworms with synthetic BmsSK and/or dsRNA that targeting BNGR-A9. In comparison to the control group, both the body weight and food consumption of *Bombyx mori* larvae notably declined on days 2 and 4 post-injection of synthetic sulfated- SK (Fig. 8A and C). Remarkably, the suppressive effect on food consumption was almost canceled by BNGR-A9 dsRNA, indicating that BmsSK functions as a satiety factor via BNGR-A9. Besides, the down-regulation of BNGR-A9 resulted in a rise in food consumption, while control dsRNA has no such effect (Fig. 8B and C).

Given that trehalose, a non-reducing disaccharide, is the primary blood sugar in insects and a crucial energy source for tissues and cells, we also explored how BmsSK signaling impacts hemolymph trehalose levels. To investigate the role of the BNGR-A9 receptor in BmsSK signaling, we measured trehalose levels in larvae after administering specific dsRNA treatments. Notably, after injecting either BNGR-A9-dsRNA or EGFP-dsRNA and subsequently treating with BmsSK, we observed a significant increase in trehalose levels in the hemolymph of larvae injected with control EGFP dsRNA. However, this increase induced by BmsSK was markedly reduced in larvae that received BNGR-A9 dsRNA, indicating the receptor's role in mediating BmsSK's effects on trehalose levels (Fig. 8D). Interestingly, glucose levels remained unchanged in both the BNGR-A9 and control dsRNA-treated silkworms, indicating a specific regulatory pathway for trehalose without affecting glucose levels (Fig. S3).

## Discussion

The SK family is pivotal in regulating various physiological processes, including feeding behavior, gut activity, heart rate, odor preference, and metabolism. While the significance of the SK signaling system in these functions is becoming clearer, the specifics of how SKs control feeding behavior are still largely unexplored. Here, we have successfully cloned and identified BNGR-A9 as the specific receptor for SK neuropeptides and explored its role in physiological regulation. Using a CRE-driven reporter system, Ca^2+^ mobilization assays, and IP3 ELISA assay, we found that the BNGR-A9 receptor is preferentially activated by *Bombyx* SKs but not by other related neuropeptides. Additionally, BNGR-A9 rapidly relocates from the cell surface to the cytoplasm upon activation by its agonists. Furthermore, exposure to SK resulted in ERK phosphorylation in both HEK293 cells and cultured silkworm brains. Taken together, our findings strongly support the idea that SKs act as functional ligands for BNGR-A9, which can now be more accurately referred to as the *Bombyx mori* SK receptor (BmSKR).

SKs were initially characterized as neuropeptides that stimulate hindgut contractions in *Leucophaea maderae*^4,5^, *Zophobas atratus*^18^, and *Locusta migratoria*^7^. Subsequent research has predominantly linked SKs to satiety regulation in insects^1,2,17,28–30^, along with their known stimulatory effects on the release of the digestive enzyme amylase from the insect digestive tract^28,31^. In our study, we used dsRNA-mediated down-regulation of BmSKR and observed an increase in food consumption, while injections of BmsSK induced weight loss. Additionally, we noted that the expression levels of SK and BNGR-A9 in midgut began to increase from the third day of the fifth instar stage and peaked on the last day. This pattern suggests that high expression levels of SK and SKR may inhibit food intake, potentially facilitating the transition from the growth phase to the spinning stage. These results emphasize the critical role of SK signaling in regulating feeding behavior and may suggest a potential role in developmental transitions of insects. Recent studies have further elucidated the role of SKs in invertebrates. For instance, the injection of Zopat-SK-1 markedly increased trehalose levels in the hemolymph, pointing to SKs' involvement in sugar regulation within insect fat body^18,32^. Our research corroborates these findings, revealing a significant rise in circulating hemolymph trehalose following BmsSK injection. Trehalose, a major storage carbohydrate in insects including *Bombyx mori*, is synthesized in the fat body from glucose and is tightly regulated by insulin-like peptides (ILPs) and adipokinetic hormone (AKH)^33^. Notably, previous studies have shown that the insulin receptor enhances SK expression and that insulin like peptide/insulin receptor regulate food intake in a SK-dependent pathway during larval stages^34^. These discoveries highlight a possible link between trehalose homeostasis and food intake regulated by SK signaling. However, the detailed mechanisms underlying the regulation of feeding behavior and circulating trehalose homeostasis by SKs still need to be further investigated.

The SK family is well conserved in invertebrates and vertebrates during evolution. Similar to the mammalian CCK/gastrin, the posttranslational sulfonation of tyrosine is also critical for the biological activities of insect SKs. Both sulfated and nonsulfated SKs were detected in the corpora cardiac/corpora allata complexes in the American cockroach, *Periplaneta americana*^24^. While sulfated sulfakinin has been reported to inhibit food intake in various insects, nonsulfated sulfakinins show little to no such activity, despite their effects on muscle activity, odor preference, and locomotion in *Drosophila*^1,21,35^. These sulfated- or non-sulfated peptides were involved in the regulation of different biological processes mainly via activating the membrane G protein-coupled receptors. In vertebrates, two CCK receptors, CCK1R and CCK2R, have been functionally characterized. CCK1R is specifically activated by the sulfated-CCK, while CCK2R is equally activated by both the sulfated or nonsulfated CCKs^36^. Similarly, in insects, two subtypes of SK receptors have been identified in *Drosophila melanogaster*^14,21^ and two in *Tribolium castaneum*^2,19^. All four receptors primarily respond to sulfated SKs. Consistent with these findings, our study demonstrates that the orphan receptor BmSKR exhibits high affinity activation by sulfated SKs, with significantly lower activity observed with nonsulfated SKs. These observations suggest the complexity and specificity of SK signaling across species and highlight the importance of posttranslational modifications in modulating their biological activities. The differential responses of sulfated and nonsulfated SKs suggest distinct regulatory roles in various physiological processes, further emphasizing the need for continued investigation into the functional diversity and mechanisms underlying SK signaling pathways.

In mammals, CCKR2 is known to couple with Gα_q_ protein, thereby activating the PLC/Ca^2+^/PKC signaling cascade, whereas CCKR1 can activate both Gα_q_ protein and Gα_s_ protein, leading to the activation of both the PLC/Ca^2+^/PKC and cAMP/PKA pathways^37,38^. However, the G protein coupling and downstream signaling cascades of insect SK receptors remain controversial. *Drosophila* SK receptor (DSK-R1) has been reported to induce calcium signaling in a PTX insensitive manner, suggesting exclusive involvement of Gα_q_ in DSKR-mediated signaling^21^. Conversely, in the red flour beetle, *Tribolium castaneum*, both SK receptors, TcSKR1 and TcSKR2, were found to be activated via Gα_s_-protein-dependent signaling pathway in transfected Sf9 cells^39^. However, another recent study employing aequorin-luminescence assay and CRE-driven luciferase reporter assay showed that both *T. castaneum* SK receptors activated both the Ca^2+^ and cyclic AMP second messenger-dependent signaling pathways^40^. Traditionally, the cyclic AMP-response element (CRE)-driven reporter gene-based functional assay has been used to determine the functional activity of Gα_s_- and Gα_i_-coupled GPCRs in mammalian cell systems. However, it has been reported that Gα_q_-coupled receptors could induce CRE-driven reporter gene transcription through a Gα_q_-dependent PKC/CREB cascade^41,42^. Indeed, our recent study also demonstrated that a Gα_q_-coupled *Bombyx* diapauses hormone receptor (BmDHR) is activated to trigger a significant increase in CRE-driven luciferase activity in a Gα_q_ inhibitor-sensitive manner^43^. In this study, we utilized the inhibitor FR900359, which specifically blocks Gα_q_ signaling by directly binding to this G protein and inhibiting the release of GDP^44–46^. Pre-incubation with FR900359 completely inhibited BmSKR-induced luciferase transcription, Ca^2+^ mobilization, and ERK phosphorylation. These findings collectively demonstrate that the *Bombyx mori* SK receptor primarily couples to the Gα_q_ protein-mediated signaling cascade.

In summary, our study has identified the orphan receptor BNGR-A9 as the specific cognate receptor for *Bombyx* neuropeptide SKs. Activation by SKs leads to coupling of BmSKR with Gα_q_ protein, initiating a PLC/Ca^2+^ signaling pathway. Pharmacological analysis indicates that the sulfate group on the Tyr residue is crucial for the full activity of *Bombyx* SKs. Additionally, quantitative RT-PCR analysis and in vivo experiments highlight the prominent role of the SK signaling system in regulating feeding behavior and circulating trehalose homeostasis. Our findings provide new insights into the mechanisms underlying BmSKR-mediated signaling in food intake control and trehalose level regulation, paving the way for further exploration of the physiological functions of the SK signaling system.

## Materials and methods

### Materials

For feeding behavior assays and qRT-PCR analyses, the silkworm strain P50 was utilized. Larvae were cultivated on fresh mulberry leaves at 25 °C in standard conditions. BmN cells were generously provided by Dr. Zhifang Zhang of the Chinese Academy of Agricultural Sciences. Synthetic neuropeptides were ordered from GL Biochem (Shanghai, China).

### Molecular cloning and plasmid construction

The entire coding region of BNGR-A9 (NM_001134272.1) was obtained by PCR and subsequently cloned into pEGFP-N1 and pCMV-Flag vectors using the primers listed in Table S1. The Flag-BNGR-A9 construct was generated for functional assays, including CRE-luciferase, calcium mobilization, and ERK phosphorylation assays, while BNGR-A9-EGFP was created for receptor visualization purposes. Additionally, for expression in the BmN cell system, the BNGR-A9 gene was inserted into the pIZT/V5-His vector. All constructs were validated by DNA sequencing.

### Cell culture and transfection

HEK293 cells were cultured with Dulbecco’s Modified Eagle’s Medium (Gibco, NY, USA) supplied with 10% Fetal bovine serum (Gibco, NY, USA) in a 37 °C humidified incubator containing 5% CO_2_. BmN cells were maintained in TC 100 insect medium (AppliChem, Darmstadt, Germany) supplied with 10% Fetal bovine serum in a 28 °C incubator. Both HEK293 and BmN cells were transfected with X-tremeGENE (Roche, Mannheim, Germany) according to the manufacturer’s instruction.

### CRE-driven cAMP luciferase assay

HEK293 and BmN cells were transfected with the different *Bombyx* receptors (Flag-BNGR-A9, -A24, -A32, or A33 for HEK293 cells, and pIZT-BNGR-A9, -A24, -A32, or A33 for BmN cells) along with the corresponding CRE-reporter plasmid^47^. When cell confluence reached 90–95%, they were treated with various concentrations of *Bombyx mori* neuropeptides, with or without forskolin. Following a 4-h incubation period, cells were lysed, and luciferase activities were determined using a firefly luciferase kit (Beyotime, Shanghai, China).

### Intracellular calcium measurement

To monitor the changes of intracellular calcium, Fura-2 AM (Dojindo, Kumamoto, Japan) was utilized as the calcium indicator, following the methodology outlined in a previous study^26^. Briefly, HEK293 cells stably expressing Flag-BNGR-A9 were exposed to various concentrations of *Bombyx mori* neuropeptides and measured using a fluorescence spectrometer (Tecan Infinite 200 PRO). Calcium levels were determined by the ratio of fluorescence excitation at 340 nm to that at 380 nm. If necessary, cells were pre-treated with U73122, FR900359, or KT5720 for 30 min prior to the initiation of the experiment. For assays involving BmN cells expressing pIZT-BNGR-A9, the procedures were conducted at 28 °C, and HBSS was replaced with HBM (Hepes-Buffered Medium: 140 mM NaCl, 5 mM KCl, 1 mM MgCl_2_, 1.2 mM Na_2_HPO_4_, 5 mM NaHCO_3_, 10 mM glucose, and 20 mM HEPES–NaOH, CaCl_2_ (1 mM), pH 6.2).

### Internalization assay

BNGR-A9-EGFP stably expressing HEK293 cells were seeded onto coverslips and incubated overnight under normal growth conditions. Subsequently, the cells were starved with fresh serum-free DMEM for 1 h and treated with either 100 nM BmsSK or BmnsSK. After a 30-min incubation period, the cells were washed three times with ice-cold PBS and fixed with 3% paraformaldehyde for 10 min at room temperature. The images were taken by a fluorescence microscopy (Leica, Germany).

### Quantitative reverse transcriptase polymerase chain reaction (qRT-PCR)

The first-strand cDNA of various tissues in fifth larval instar P50 silkworm was synthesized with PrimeScript 1st Strand cDNA Synthesis Kit (Takara, Japan) using an oligo(dT)18 primer and 1 μg total RNA template in a 20 μl reaction, following the instructions. Subsequently, qRT-PCR analysis for *bngr-a9* and *sk* genes were performed with primers listed in Table S1 using SYBR ExTaq Premix (Takara, Japan). Melt curve analysis was further performed at the end of the PCR cycles to confirm the specificity of primers. Relative quantification was performed via the comparative 2^−ΔΔCT^ method. Specifically, the Ct values of the target genes were normalized to the geometric average of the Ct value of reference genes GAPDH and Actin A3. The experiments were performed in duplicate with three biological replicates.

### In vitro dsRNA synthesis and injection

Double-stranded RNAs were synthesized using MEGAscript RNAi T7 Kit (Ambion, CA, USA) following the manufacturer’s instructions. Templates for in vitro transcription were generated by PCR using the primers containing the T7 polymerase promoter sequence at their 5′ ends, as listed in Table S1. For each 20-μl reaction, 1 μg PCR product was added as the template. Following transcription, the reaction mixture was purified by phenol/chloroform extraction and ethanol precipitation. The resulting dsRNA products were diluted with nuclease-free water to achieve the desired concentration (final volume of 2–5 μl).

### Feeding behavior assay

Synchronized larvae of the first day of 5th instar were chosen for feeding behavior assay. Silkworms were starved for 12 h to standardize the starting level. To knock down *bngr-a9*, 5 μg dsRNA/BNGR-A9 or dsRNA/EGFP was injected into the abdomen of the larvae at the indicated time using a 10 μl microsyringe. For silkworms, both treated and untreated with dsRNA, synthetic SK peptide (at a final concentration of 10 nM) was injected daily starting from the second day of the fifth instar. For each trial, twenty synchronized P50 larvae were used. After injection, the silkworm larvae were fed with enough mulberry leaves three times a day and maintained under standard conditions. Larval body weights and food consumption were measured every other day until pupation.

### Determination of trehalose of hemolymph

Silkworm larvae were anesthetized by cooling on ice, after which hemolymph was collected using a micropipette from an incision made at the abdominal leg. Approximately 5 mg of phenylthiourea (to achieve a final concentration of 2.5% w/v) was added to the hemolymph in a 1.5 ml Eppendorf tube to inhibit clotting. Hemolymph samples from 10 insects were then centrifuged at 12,000 rpm for 10 min at 4 °C to sediment any particulates. For trehalose measurement, the hemolymph underwent pretreatment with trehalase (Megazyme, Chicago, US), following the manufacturer’s protocol. Subsequently, the sugar concentration in the hemolymph was assessed using a glucose oxidase–peroxidase kit (Shanghai Rongsheng Biotech, China).

### ERK phosphorylation and western blot

HEK293 cells with Flag-BNGR-A9 were exposed to 100 nM BmsSK for different durations and then lysed using RIPA buffer (Beyotime, China). For ex vivo assays, brains from 5th instar silkworm larvae were treated with BmsSK (100 nM) for 1, 2, or 8 h, either with a pre-treatment of Gα_q_ inhibitor FR900359 for 30 min or without it. After treatment, samples were lysed, homogenized, and their protein concentrations were determined with a BCA protein kit (Takara, Japan). For western blot analysis, equal amounts of protein lysate were used. Protein expression was analyzed using specific primary antibodies: rabbit monoclonal antibodies for total and phosphorylated ERK from Cell Signaling Technology (MA, USA).

### Data analysis

Data analysis was conducted using GraphPad Prism (San Diego, CA), with results presented as mean ± SEM. Statistical significance was assessed through Student’s t-test or two-way ANOVA with multiple comparisons, with p values below 0.05 denoted as significant (*p < 0.05, **p < 0.01, ***p < 0.001). Dose–response curves were generated via non-linear curve fitting. Adobe Photoshop was used to process images, ensuring uniform brightness and contrast settings across all conditions. All images and data provided represent findings from a minimum of three independent experiments.

### Supplementary Information

Supplementary Information.

## Data Availability

The data that support the findings of this study are available from the corresponding author upon reasonable request.

## Abbreviations

GPCRs: G protein-coupled receptors
BNGR: *Bombyx mori* Neuropeptide G protein-coupled receptor
BmsSK: Bombyx mori sulfated-sulfakinin
BmnsSK: *Bombyx mori* Non-sulfated-sulfakinin
CRE: cAMP response element
qRT-PCR: Quantitative Reverse Transcriptase Polymerase Chain Reaction
PLC: Phospholipase C
PKA: Protein kinase A
