# Supplementary material for: Identification of sulfakinin receptor regulating feeding behavior and hemolymph trehalose homeostasis in the silkworm, Bombyx mori
Source: Sci Rep. 2024 Jun 20;14:14191. doi: 10.1038/s41598-024-65177-z (PMC11190223; doi:10.1038/s41598-024-65177-z)
Supplement: Supplementary file 1 — Supplementary Information. [file 41598_2024_65177_MOESM1_ESM.pdf]

Identification of sulfakinin receptor regulating feeding behavior and hemolymph trehalose homeostasis in the silkworm, *Bombyx mori*

Jiajing Lan[1#], Qi Wu[2#], Nan Huang[3], Hong, Zhang[1], Yuanfei, Yang[1], Linjie Chen[1], Naiming Zhou[4*], Xiaobai He[1*]

[1]School of Laboratory Medicine and Bioengineering, Hangzhou Medical College, Hangzhou, 311399, China;

[2]College of Biotechnology, Jiangsu University of Science and Technology, Zhenjiang, Jiangsu, 212018, China;

[3]Department of Clinical laboratory, the First People's Hospital of Lin'an District, Hangzhou, Zhejiang, 311399, China;

[4]Institute of Biochemistry, College of Life Sciences, Zijingang Campus, Zhejiang University, Hangzhou, Zhejiang, 310058, China.

[#] Both authors contributed equally to this work.

[*]To whom correspondence should be addressed:

Xiaobai He: School of Laboratory Medicine and Bioengineering, Hangzhou Medical College, Hangzhou, China, shining0206@163.com;

Naiming Zhou: Institute of Biochemistry, College of Life Sciences, Zijingang Campus, Zhejiang University, znm2000@zju.edu.cn

Supplementary Materials

Material and methods

1. Measurement of cAMP accumulation

Cells were seeded in 24-well plates and incubated overnight. Prior to treatment, cells were pre-treated with the phosphodiesterase inhibitor IBMX (300 μM) for 1 hour. Subsequently, cells were treated with different *Bombyx* neuropeptides for 15 minutes. The reaction was terminated by removing the medium, followed by the addition of ice-cold PBS and a single wash. Cell lysis was performed, and cAMP formation was assessed using a competitive binding technique based on an enzyme-linked immunosorbent assay (ELISA) (Parameter cAMP assay, R&D, Minneapolis, USA) following the manufacturer's instructions. The results were expressed as the fold change in cAMP concentration compared to the control concentration in the supernatants.

2. Determination of Glucose of hemolymph

Silkworm larvae were anesthetized by cooling on ice, after which hemolymph was collected using a micropipette from an incision made at the abdominal leg. Approximately 5 mg of phenylthiourea (to achieve a final concentration of 2.5% w/v) was added to the hemolymph in a 1.5 ml Eppendorf tube to inhibit clotting. Hemolymph samples from 10 insects were then centrifuged at 12,000 rpm for 10 minutes at 4 °C to sediment any particulates. The glucose concentration in the hemolymph was determined using a glucose oxidase-peroxidase kit (Shanghai Rongsheng Biotech, China).

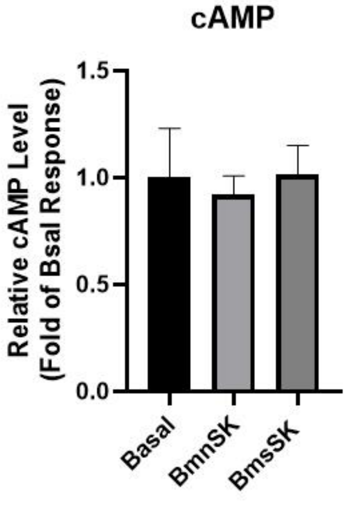

Fig. S1. Accumulation of cAMP in BNGR-A9 expressing HEK293 cells in response to *Bombyx* sulfakinins. Cells were pretreated with the PDE1 inhibitor IBMX for 1 h followed by stimulation with 1μM BmnsSK or BmsSK for 15 min, and the cAMP level was determined by the cAMP immunoassay detection kit.

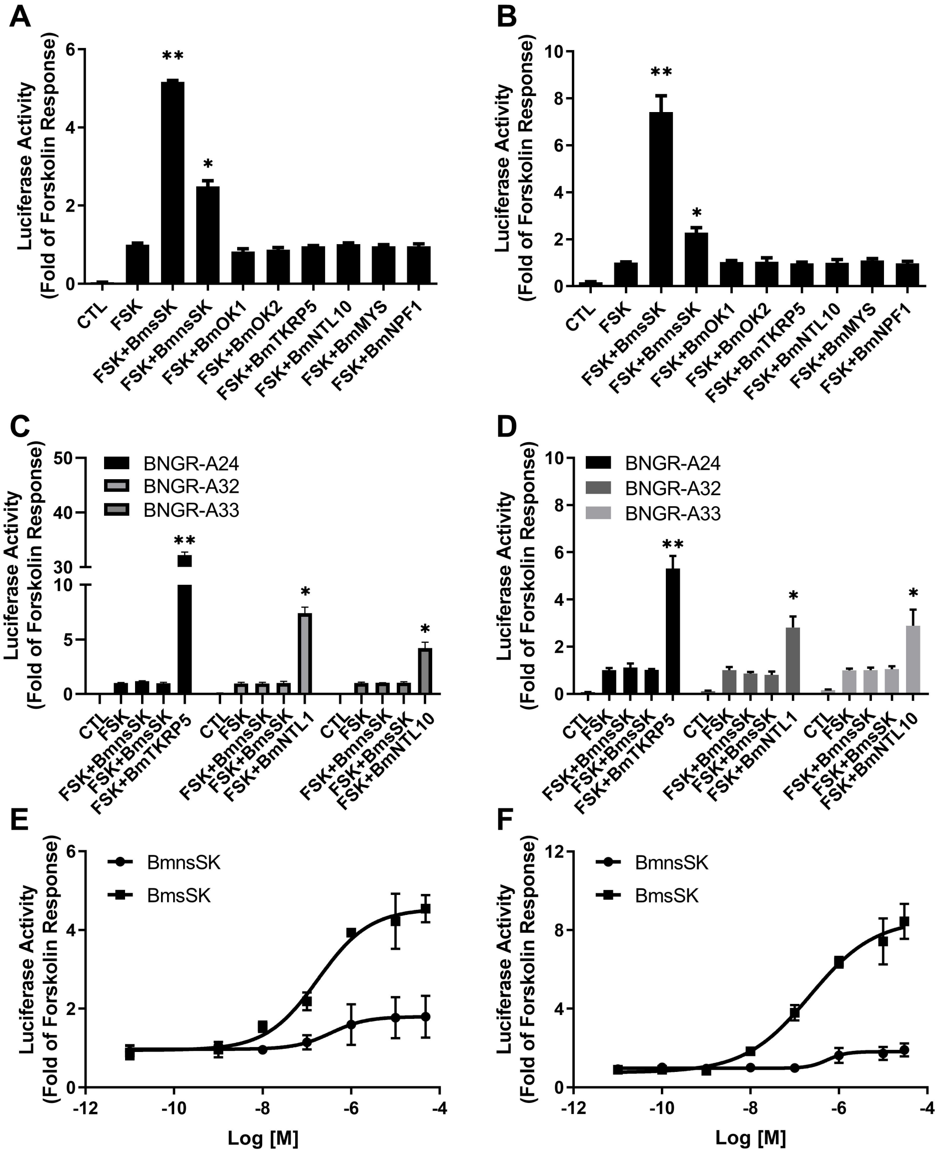

Fig. S2. BNGR-A9 is a specific *Bombyx mori* sulfakinin peptides receptor. HEK293 cells (A) or BmN cells (B) transfected with BNGR-A9 and the corresponding reporter gene pCRE-Luc were treated with 1 μM of different *Bombyx mori* neuropeptides under the condition of 5 μM forskolin, and responses were normalized against the luciferase activity of forskolin. Abbreviations: SK, sulfakinin; SSK, sulfated-sulfakinin; TKRP, tachykinin-related peptide; NTL, natalisin; OK, orcokinin; MYS, myosuppressin; NPF1, neuropeptide F1. **C** and **D**, HEK293 cells (C) and BmN cells (D) transiently transfected with BNGR-A24, BNGR-A32 or BNGR-A33 and the reporter gene pCRE-Luc, and then treated with 1 μM of *Bombyx* sulfakinins or their corresponding ligands under the condition of 5 μM forskolin, followed by detection of luciferase activities. **E** and **F**, Dose-response curves of HEK293 cells (E) and BmN cells (F) stably expressing BNGR-A9 and the corresponding reporter gene pCRE-Luc treated with different concentration of *Bombyx* sulfakinins. Data were analyzed by using a Student's t-test ((*p < 0.05; **p < 0.01). All data were taken from at least three independent experiments.

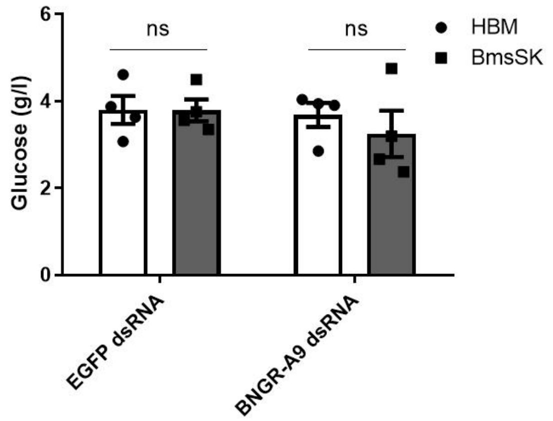

Fig. S3. Effect of dsRNA and sulfated-sulfakinin SK on the glucose level in the hemolymph. Twenty-four hours after dsRNA injection, silkworm larvae were injected with BmsSK (final 10 nM) or the same volume of HBM, then hemolymph was collection 24 hours later and glucose level were determined as described in Material and Method. All data were taken from at least three independent experiments, and data were analyzed using a Student's t-test (*$p < 0.05$).

Figure 7A and B

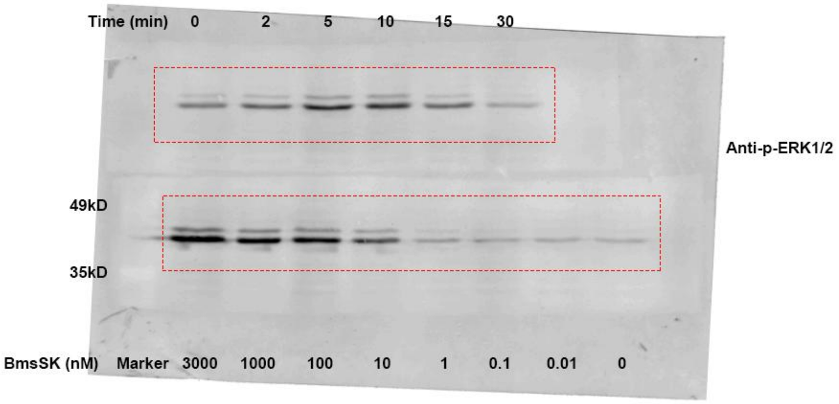

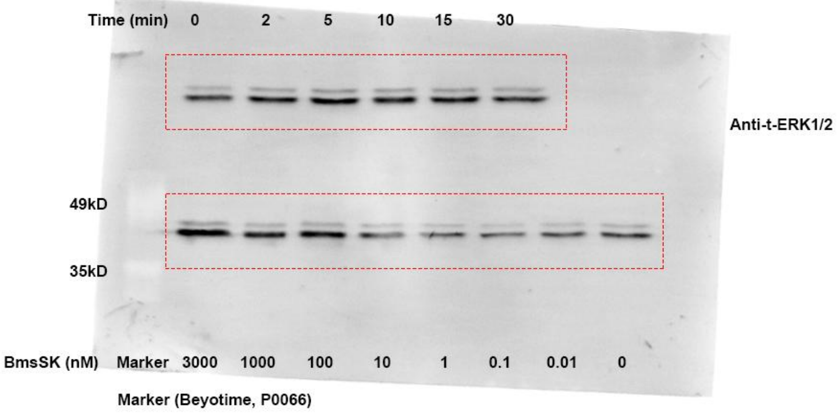

Figure 7C

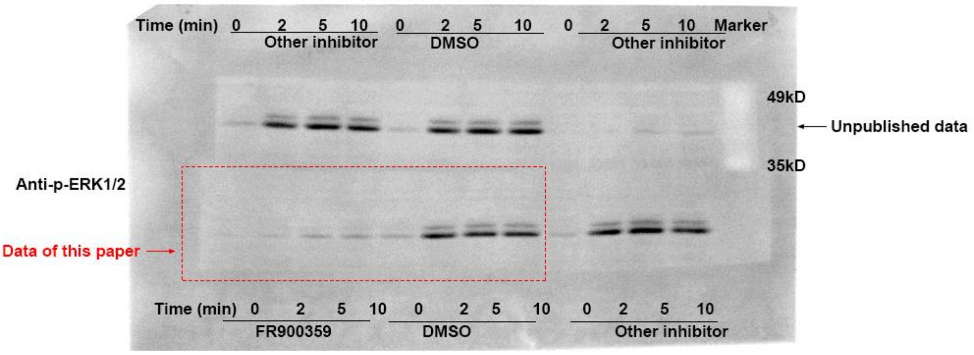

Marker (Beyotime, P0066)

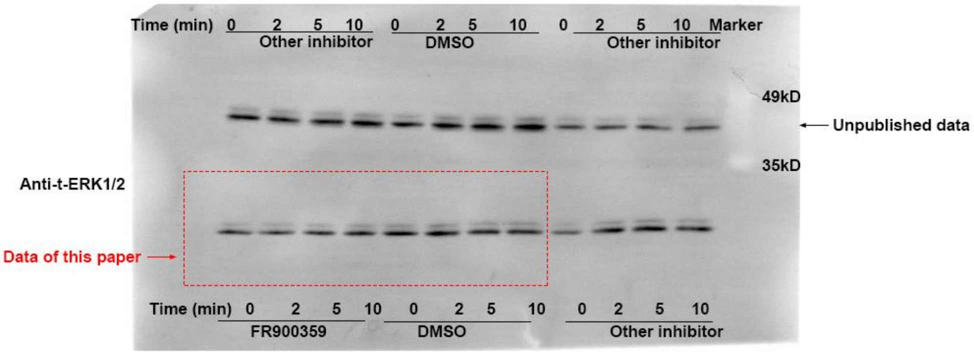

Figure 7D

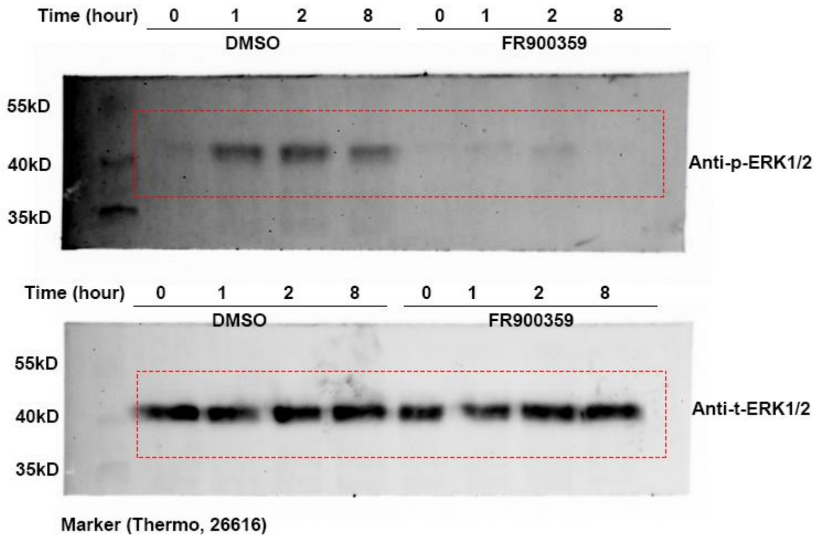

Fig. S4 Raw images of the data from Figure 7.

Table S1. List of primers used in this study

Primers for Flag-BNGR-A9	5'-GAAGATCTATGAATGATCCGGATATAAGCTAC-3'
	5'-GCTCTAGACTAAACACAATCCTTCCCCTCC-3'
Primers for BNGR-A9-EGFP	5'-CCGCTCGAGGCCACCATGAATGATCCGGATATAAGCTAC-3'
	5'-GGGGTACCGTAACACAATCCTTCCCCTCCAGTC-3'
Primers for qRT-PCR of *bngr-a9*	5'-CGAATCAAACGAACCAGAAGAAACC-3'
	5'-TGGGTAGACCTTACGAAATGCCTG-3'
Primers for qRT-PCR of *sk*	5'-TAACTTTCTGCGTGTGTTGCTG-3'
	5'-TCCTCGGATAACCCTGCTTCTA-3'
Primers for qRT-PCR of *Bombyx* Actin A3	5'-CGTTCGTGATATCAAGGAGAAGCT-3'
	5'-TCCATACCCAAGAACGAGGGTTG-3'
Primers for qRT-PCR of *Bombyx* GAPDH	5'-CATTCCGCGTCCCTGTTGCTAAT-3'
	5'-GCTGCCTCCTTGACCTTTTGC-3'
Primers for BNGR-A9 dsRNA template	5'-TAATACGACTCACTATAGGGAACAGCTCGCTGCTGAGAAT-3'
	5'-TAATACGACTCACTATAGGGACAATCCTTCCCCTCCAGTC-3'
Primers for EGFP dsRNA template	5'-TAATACGACTCACTATAGGGATGGTGAGCAAGGGCGAGGAG-3'
	5'-TAATACGACTCACTATAGGGCTTGTACAGCTCGTCCATGC-3'
